# Synthesis and Comparison of In Vitro Leishmanicidal Activity of 5-(Nitroheteroaryl)-1,3,4-Thiadiazols Containing Cyclic Amine of Piperidin-4-ol at C-2 with Acyclic Amine Analogues against Iranian Strain of *Leishmania major* (MRHO/IR/75/ER)

**Published:** 2017-03-14

**Authors:** Azar Tahghighi, Alireza Foroumadi, Susan Kabudanian-Ardestani, Seyed Mohammad Amin Mahdian

**Affiliations:** 1 Malaria and Vector Research Group, Biotechnology Research Center, Pasteur Institute of Iran, Tehran, Iran; 2 Faculty of Pharmacy and Pharmaceutical Sciences Research Center, Tehran University of Medical Sciences, Tehran, Iran; 3 Department of Biochemistry, Institute of Biochemistry and Biophysics, University of Tehran, Tehran, Iran

**Keywords:** *Leishmania major*, Thiadiazole, Nitrofuran, Nitrothiophen, Superimpose

## Abstract

**Background:**

Cutaneous Leishmaniasis (CL) is endemic in many tropical and subtropical regions of the world. Due to the prolonged duration of therapy, adverse effect and resistance to current drugs in the treatment of CL, the discovery of novel, efficient, and safe leishmanicidal drugs is required. The aims of the present study was to synthesis of new compounds based on the active compounds of 5-(5-nitrofuran-2-yl)- and 5-(5-nitrothiophen-2-yl)-1,3,4-thiadiazole bearing the linear amino alcohol of 3-aminopropan-1-ol in the C-2 position of thiadiazole ring and evaluation of their activity against the promastigote and amastigote forms of *Leishmania major*.

**Methods:**

Reaction between the solution of 5-(5-nitro heteroaryl)-2-chloro-1,3,4-thiadiazole and piperidin-4-ol in absolute ethanol was performed and the resulting products were evaluated against promastigotes form of *L. major* with MTT assay and amastigote form of *L. major* in murine peritoneal macrophages. In addition, the toxicity of these compounds was assessed against mouse peritoneal macrophages with MTT assay.

**Results:**

New synthetic compounds 5a-b showed moderate in vitro antileishmanial activity against *L. major* promastigotes with IC_50_ values of 68.9 and 27μM, respectively. These compounds have also demonstrated a good antiamastigote activity in terms of amastigote number per macrophage, the percentage of macrophage infectivity and infectivity index.

**Conclusion:**

Novel cyclic compounds 5a-b were synthesized and exhibited less antipromastigote and antiamastigote activity compared to linear analogues.

## Introduction

Leishmaniasis is an endemic parasitic disease and a major public health problem in more than 90 countries around the world (CDC 2013). It is estimated that 1.3 million people are affected by this disease annually and the overall population at risk is 310 million people (WHO 2010). There are four forms of leishmaniasis: Visceral Leishmaniasis (VL) or kala azar, which is typically lethal if left untreated, Mucocutaneous Leishmaniasis (MCL), which is a mutilating disease, Diffuse Cutaneous Leishmaniasis (DCL) related to defective immune system and Cutaneous Leishmaniasis (CL) that produces serious skin lesions (Singh and Sivakumar 2004). The current chemotherapy against all forms of leishmaniasis including parental pentavalent antimonial compounds remains the primary therapy, followed by making use of other drugs like amphotericin B, paramomycin, pentamidine and orally miltefosine (Monzote 2009). However, making use of them is limited owing to toxicity and the high cost of treatment (Chawla and Madhubala 2010, Tiuman et al. 2011). In addition, the development of clinical resistance and an increasing incidence of leishmaniasis/AIDS co-infection in some regions is a serious health problem (Molina 2003). As such, the design of new, efficient, cheap and safe drugs for the treatment of leishmaniasis is imperative.

**Fig. 1.**
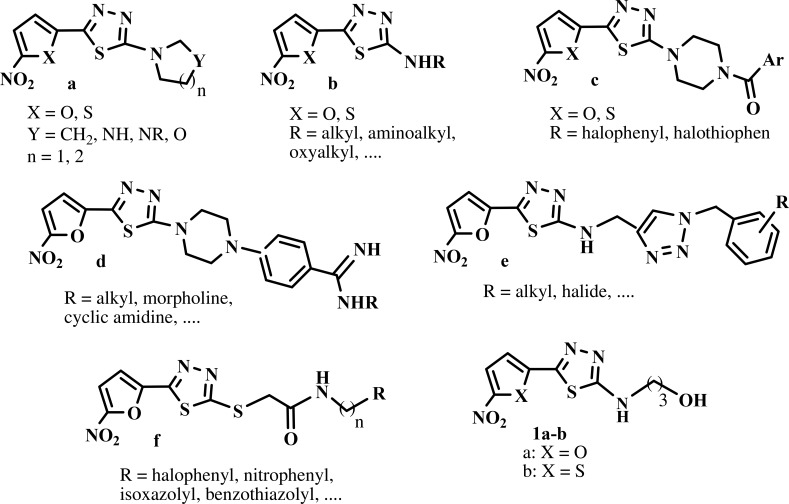
General structures of antileishmanial compounds (a) bearing a cyclic amine (b) containing acyclic amine (c-f) with aromatic substitutions and (1a-b) containing hydroxypropyl amine at the C-2 position of thiadiazole ring

5-(nitroheteroaryl)-1,3,4-thiadiazole containing a cyclic (a) and acyclic (b) amine in C-2 position, depending upon the type of substituent, have shown a promising antileishmanial activity (Fig. 1). Furthermore, different substitutions in C-2 position in 1,3,4-thiadiazole ring have been able to affect potency and physicochemical properties. On this basis, to achieve a novel anti-leishmanial agent, the new derivatives were synthesized to introduce other groups on C-2 amine of thiadiazole ring such as, 4-aroylpiperazine segment (c), piperazinyl-linked benzamidines substituent (d), N-[(1-benzyl-1*H*-1,2,3-triazol-4-yl)methyl] moiety (e) and various 2-thioacetamides substituent (f) (Fig. 1). These different attachments to 1,3,4-thiadiazole rings causes changes the bioresponses, depending upon the type of substituent and position of attachment (Foroumadi et al. 2008, Tahghighi 2011, 2012, 2013, Marznaki 2013, Vosooghi 2014). Recently, given the importance of this position, several 5-(5-nitrofuran-2-yl)- and 5-(5-nitrothiophen-2-yl)-1,3,4-thiadiazoles possessing acyclic amines in the C-2 position of thiadiazole ring were synthesized and theirs in vitro activity was evaluated against the promastigotes and amastigotes forms of *L. major*. Two compounds 1a-b with linear substitution of 1-propanol amine exhibited a promising antileishmanial activity against *L. major* promastigotes (Fig. 1) (Tahghighi et al. 2013).

Concerning these findings and simple reaction for preparation these compounds, the aim of this study was to synthesize cyclic analogues of these linear amino alcohols, examine anti-leishmanial activity against promastigote and amastigote forms of *L. major* and evaluate their cytotoxicity activity on macrophages.

## Materials and Methods

### Chemistry

All chemical reagents and solvents were purchased from Merck Company and used without further purification. The Key intermediate 2-chloro-1,3,4-thiadiazole 4a-b was prepared by starting from 5-nitrofurfurilidine diacetate or 5-nitrothiophene-2-carboxaldehyde (Tahghighi et al. 2011, 2012, 2013, Marznaki 2013, Vosooghi 2014). The melting points of compounds were determined using a Kofler hot-stage apparatus. The IR spectra were obtained on a Shimadzu 470 spectrophotometer using KBr dicks. ^1^H NMR spectra were recorded on a Varian unity 500 and 400 spectrometer and chemical shifts (δ) were reported in parts per million (ppm) relative to tetramethylsilane (TMS) as an internal standard. The mass spectra were run on an Agilent 6410 LC-MS or a FiniganTSQ-70 spectrometer (Finigan, USA) at 70 eV. Merck silica gel 60 F254 plates were used for analytical TLC. Compounds 1a-b has been described in our previous work (Tahghighi et al. 2013).

### General procedure for the synthesis of compounds 5a-b

**Fig. 2.**
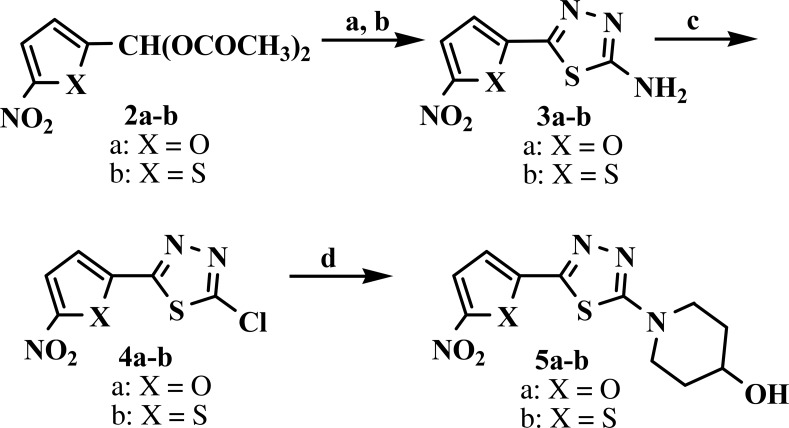
Synthetic route to compounds 5a-b. Reagents and conditions: a) thiosemicarbazide, EtOH, HCl, reflux, b) NH_4_Fe(SO_4_)_2_.12H_2_O, H_2_O, reflux; c) NaNO_2_, HCl, Cu, d) piperidin-4-ol, EtOH, reflux

A solution of 2-chloro-1,3,4-thiadiazole 4a-b (1.1mmol) and appropriate cyclic amine of piperidin-4-o l (1mmol) in absolute ethanol (7ml) was refluxed until the reaction was completed (4h). Then, the solvent was evaporated under reduced pressure and the residue was purified using silica gel column chromatography, eluting with appropriate solvent (Fig. 2).

### 1-(5-(5-nitrofuran-2-yl)-1,3,4-thiadiazol-2-yl)piperidin-4-ol (5a)

The resulting product was purified using a short silica gel column eluting with CH_3_COOC_2_H_5_ followed by CH_3_COOC_2_H_5_ containing 2% methanol. The compound was obtained as a yellow solid (Yield: 78%). Mp 147.7–149 °C. IR (KBr, cm^−1^): 3414, 1734, 1535, 1497 and 1344. ^1^H NMR (500 MHz, DMSO-*d*_6_) δ: 7.84 (d, 1H, *J*= 3.25 Hz, furan), 7.35 (d, 1H, *J*= 3.25 Hz, furan), 3.80 (m, 3H, CH_2_ and CH), 3.41 (t, 2H, CH_2_), 3.33 (brs, 1H, OH), 1.85 (m, 2H, CH_2_), 1.51 (m, 2H, CH_2_). MS (ESI): 296.8 [M + H^+^].

### 1-(5-(5-nitrothiophen-2-yl)-1,3,4-thiadiazol-2-yl)piperidin-4-ol (5b)

The resulting product was purified using a short silica gel column eluting with CH_3_COOC_2_H_5_ followed by CH_3_COOC_2_H_5_ containing 2% methanol. The compound was obtained as an orange solid (Yield: 73%). mp160–162 °C. IR (KBr, cm^−1^): 3298, 1736, 1536, 1494 and 1354. ^1^H NMR (400 MHz, CDCl_3_) δ: 7.86 (d, 1H, J= 4.4 Hz, thiophen), 7.16 (d, 1H, J= 4.4 Hz, thiophen), 4.05 (brs, 1H, OH), 3.91 (m, 3H, CH_2_ and CH), 3.49 (t, 2H, J= 9.3 CH_2_), 2.03 (m, 2H, CH_2_), 1.73 (m, 2H, CH_2_). MS (ESI): 312.8 [M+ H^+^].

### Antileishmanial activity against *Leishmania major* promastigotes

The antileishmanial activity of compounds 5a-b was performed using MTT assay (Dutta et al. 2005). The promastigote form of parasite (vaccine strain MRHO/IR/75/ER, obtained from Pasteur Institute, Tehran, Iran) was grown in blood agar cultures at 25 °C. The growth curve of the *L. major* strain was determined daily under light microscope and counting in a Neuberger’s chamber. Then, parasites (2× 10^6^/ml) in the logarithmic phase were incubated with different concentrations (12.5, 25, 50 and 75μg/ml) of test compounds for 24 h at 25 °C. A negative control (DMSO without any test compounds with culture medium), and positive control (with Glucantime and Fluconazole) were used on same plate. After incubation, the media was renewed with 100μg/well of MTT (0.5mg/ml) and plates were further incubated for 4 h at 37 °C. Then, the plates were centrifuged (2000rpm× 5min× 4 °C) and the obtained pellets were dissolved in 200μL of DMSO. The optical density (OD) of samples due to cleavage of the tetrazolium salt MTT [3-(4,5-dimethylthiazol-2-yl)-2,5-diphenyl tetrazolium bromide] into a colored product formazan by the parasite was measured by ELISA plate reader at a wavelength of 492nm. Two or more independent experiments in triplicate were performed for each compound. The IC_50_ values (the concentration required to inhibit 50% growth of promastigotes after 24h) were calculated by linear regression analysis, expressed in mean ± SD.

### Antileishmanial activity against *Leishmania major* amastigotes

Compounds 5a-b was evaluated for their activity against amastigote form of *L. major* in murine peritoneal macrophages. Briefly, the abdominal cavity of BALB/c mice was torn then washed to inject 5 ml of phosphate buffered saline (PBS) by a sterile syringe and collected cells. The cell suspension was centrifuged (2000rpm× 10min× 4 °C). Mouse peritoneal macrophages were plated in RPMI 1640 supplemented with 10% of heat-inactivated fetal bovine serum, 2mM glutamine, 100U/ml penicillin (Sigma) and 100μg/ml streptomycin. Macrophages were placed on sterile glass cover slips in 24-well plates (1× 10^6^/well). After 1h, non-adherent cells were removed by washing with RPMI 1640, the stationary phase promastigotes in RPMI 1640 were added (2×10^6^ parasites/well, three parasites/macrophage) to macrophage monolayer and the plates were kept at 37 °C in a CO_2_ incubator for 2h. Extracellular parasites were removed by washing and then new media containing IC_50_ concentration of the drug were added. Two sets of experiments were carried out for each compound at 24h. Following these procedures, cells were fixed with methanol, stained with Giemsa stain (Sigma) and the infectivity index was determined by multiplying the percentage of macrophages that had at least one intracellular parasite by the average number of intracellular parasites per infected macrophage (100 cells were examined/well) (Tanaka et al. 2007).

### Toxicity against macrophages

The toxicity of compounds 5a-b was assessed against mouse peritoneal macrophages plated in 96-well plates at 2× 10^5^ cells/well. After cell adherence, the medium was removed and replaced by the media containing different concentrations of each compounds. The plates were incubated for 24 h at 37 °C in a humidified incubator with 5% CO_2_. DMSO without any test compounds was used as a negative control. Cell viability was determined by MTT colorimetric assay (Kiderlen et al. 1990). Two independent experiments in triplicate were performed for determination of toxicity of each compound. The CC_50_ (cytotoxic concentration for 50% inhibition) were calculated by linear regression analysis.

### Overlay study of compounds

The 3D structures of the ligands of Table 1 were drawn using HyperChem software (version 7.0) and subsequently energy minimized using semiemperical method, AM1 level of theory. Then, energy minimized molecules were superimposed using atoms selection. In this way, one of the molecules was opened and atoms selected, then the second molecule was opened and the same atoms selected and tow molecules were superimposed by Overlay option of HyperChem program.

**Table 1.** In vitro antileishmanial activity of thiadiazole derivatives 1a-b and 5a-b against promastigote form of *Leishmania major*

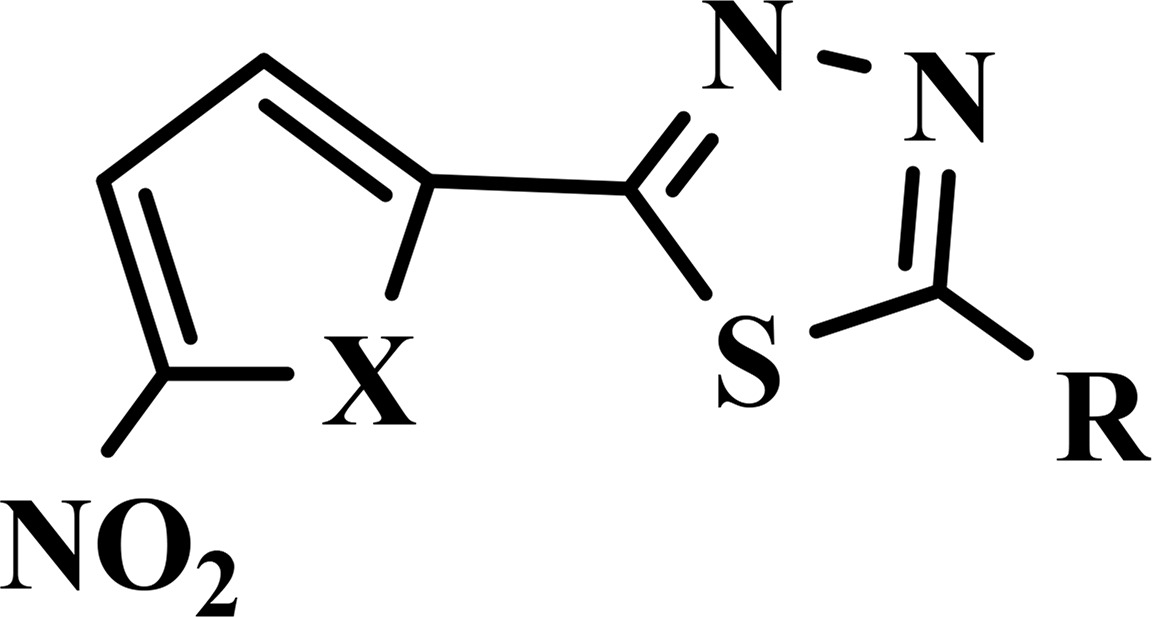					
**Compounds**	**R**	**X**	**Anti-promastigote activity IC_50_ (μM)**	**Cytotoxicity CC_50_ (μM)^a^**	**SI^b^**
**1a**	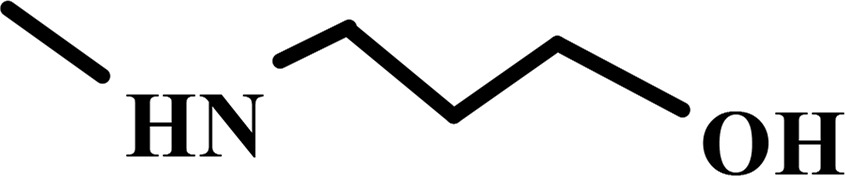	O	18 ± 0.2	62.33	3.46
**1b**	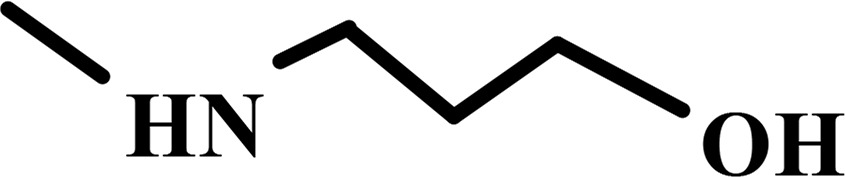	S	3 ± 0.41	42.16	14.05
**5a**	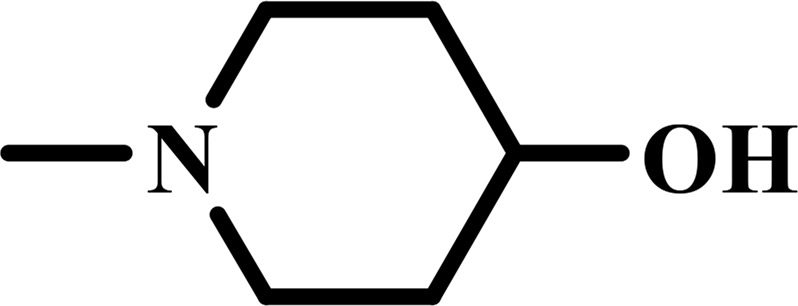	O	68.9 ± 0.107	78.54	1.14
**5b**	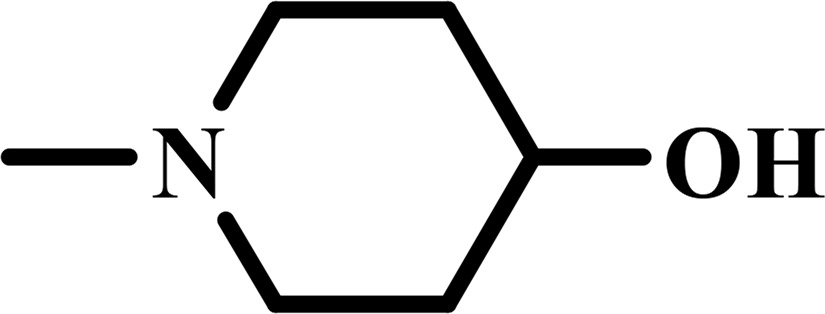	S	27 ± 0.12	63.55	2.35
**Glucantime**	-	-	68.44^c^	-	-
**Fluconazole**	-	-	941.1 ± 4.98	-	-

a Cytotoxicity was evaluated against mouse peritoneal macrophages.

b Selectivity index (SI) CC_50_/IC_50_.

c The IC_50_ of Glucantime was in mM.

## Results

### Chemistry

The intermediate 2-chloro-1,3,4-thiadiazole 4a-b was obtained from 5-nitrofurfurilidine diacetate or 5-nitrothiophene-2-carboxaldehyde [7–12]. The target compounds 5a-b were synthesized in high yield by the reaction of 5-(5-nitro heteroaryl)-2-chloro-1,3,4-thiadiazole 4a-b and appropriate piperidin-4-ol in refluxing absolute ethanol (Fig. 2). All of the synthesized compounds were characterized by IR, ^1^H NMR and LC-MS.

### Biological activity

We synthesized novel 5-(nitroheteroaryl)-1,3,4-thiadiazols containing acyclic amines in C-2 and evaluated antileishmanial activity of these compounds against the promastigote and amastigote stage of *L. major*. The compounds of 3-(5-(5-nitrofuran-2-yl)-1,3,4-thiadiazol-2-ylamino) propan-1-ol 1a and 3-(5-(5-nitrothiophen-2-yl)-1,3,4-thiadiazol-2-ylamino) propan-1-ol 1b showed IC_50_ values of 18±0.2 and 3±0.41μM, respectively, against the promastigotes form of *L. major* and, in contrast, the analogue of 5-nitrothiophen 1b showed 6-fold more potent than its 5-nitrofuran counterpart 1a. This finding indicated that different responses take place due to O/S replacement in the scaffold.

The previous compounds 1a-b was also evaluated against the amastigotes form of *L. major*. These compounds decreased the number of intracellular amastigotes per macrophage, the percentage of macrophage infectivity and infectivity index compared with the control group. The in vitro cytotoxic activity of the compounds 1a-b demonstrated toxicity against mouse peritoneal macrophages (CC_50_ values of 62.33 and 42.16μM, respectively). Furthermore, the compound 1b displayed the highest selectivity index (SI= 14.05) (Table 1).

**Fig. 3.**
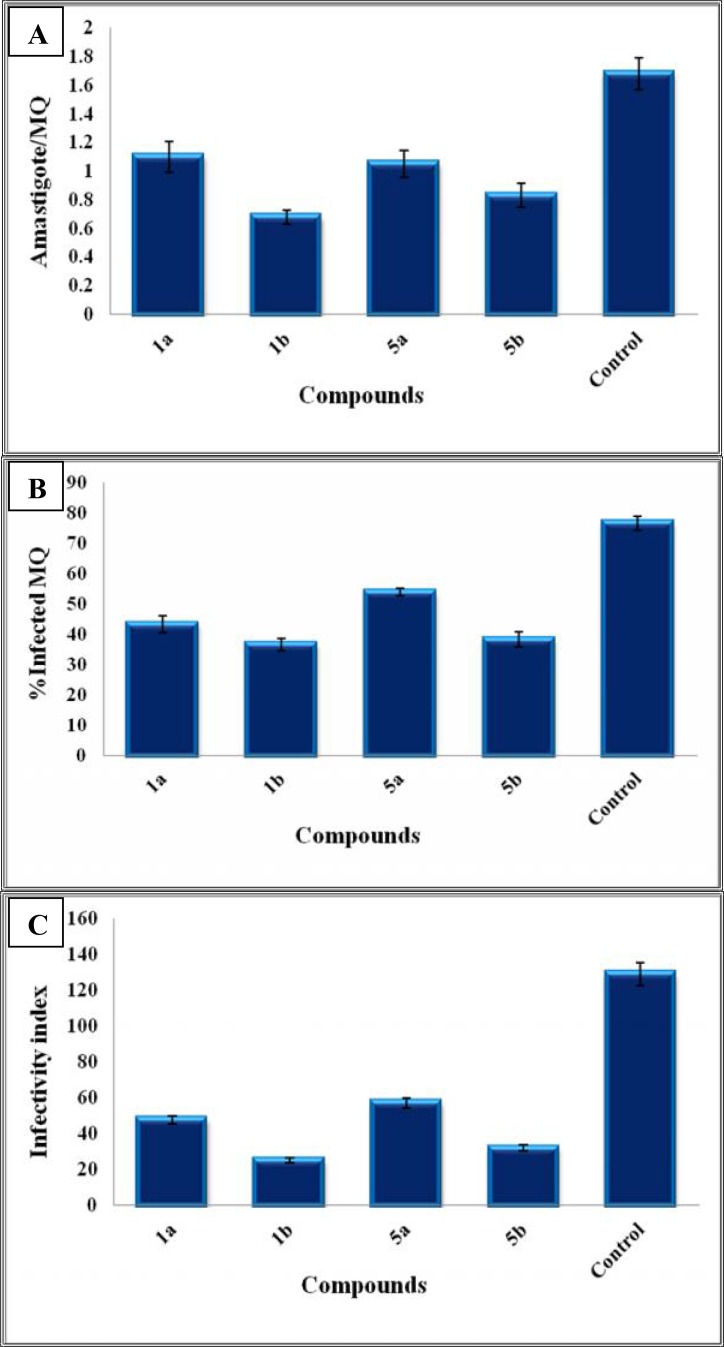
The in vitro activity of selected compounds against intramacrophage amastigotes of *Leishmania major*. (A) The mean number of amastigotes per macrophage after treatment with selected compounds for 24h. (B) The percentage of infected macrophages after treatment. (C) Infectivity index of macrophages cultured 24 h in presence of selected compounds. The infectivity index was determined by multiplying the percentage of macrophages that had at least one intracellular parasite by the average number of intracellular parasite per infected macrophage (100 cells were examined/well).

Moreover, we synthesized two cyclic compounds 5a-b similar to these linear compounds and evaluated their activity against the promastigote and amastigote forms of *L. major*. Fluconazole and Meglumine antimonate (Glucantime^®^) were used as reference drugs. These compounds have a medium anti-promastigote activity with IC_50_ values less than 70μM. Furthermore, these compounds were evaluated for their activity against amastigote form of *L. major* in murine peritoneal macrophages (Fig. 3) and thiophen analogues 1b and 5b exhibited higher activity against amastigotes as showed by amastigote number per macrophage, the percentage of macrophage infectivity and infectivity index compared with furan analogues 1a and 5a (Fig. 3A, 3B and 3C, respectively).

In addition, the CC_50_ values for these compounds 5a-b against mouse peritoneal macrophages were determined using MTT assay (Table 1). They were toxic to macrophages (CC_50_< 80 μM) and that the compound 1a had the highest selectivity index (SI= 14.05).

### Overlay study

**Fig. 4.**
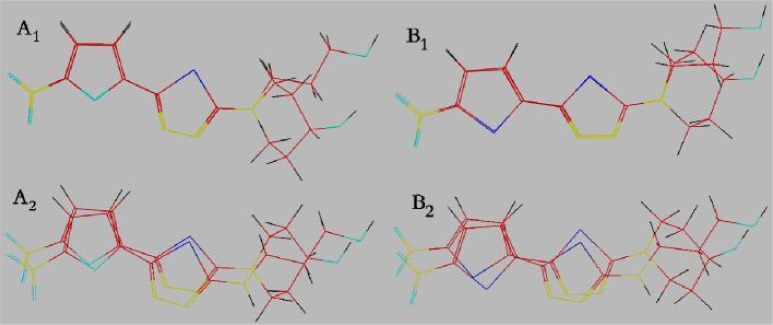
**A_1_:** Overlay between compounds 1a and 5a without hydroxyl selection. **A_2_:** Overlay between compounds 1a and 5a with hydroxyl selection. **B_1_:** Overlay between compounds 1b and 5b without hydroxyl selection. **B_2_:** Overlay between compounds 1b and 5b with hydroxyl selection.

The superimposition studies of the target compounds 5a-b and lead molecules 1a-b in their energy-minimized conformations and without hydroxyl group’s selection revealed that the thiophen or furan and thiadiazole ring, nitro group and amine group in C-2 position of thiadiazole ring at these compounds overlaid completely on each other whereas hydroxyl groups located in different positions (Fig. 4 A_1_, B_1_). The superimposition of these compounds with hydroxyl group selection showed that there was a weak overlay between target and lead molecules (Fig. 4 A_2_, B_2_).

## Discussion

The cyclic analogues of 5-(5-nitrofuran-2-yl)- and 5-(5-nitrothiophen-2-yl)-1,3,4-thiadiazole-2-amines bearing piperidin-4-ol at the C-2 position of thiadiazole ring were synthesized and evaluated in vitro against promastigote and amastigote forms of *L. major*. These novel compounds, which were compared with their linear analogues, exhibited less antileishmanial activity against promastigote form of *L. major.*

Our previous studies demonstrated that the C-2 substituent in 5-(nitroheteroaryl)-1,3,4-thiadiazoles is the most flexible site for chemical change and is an area where it determines the potency and physicochemical properties of these synthetic compounds. Accordingly, several series of these derivatives were synthesized by our research group with different linkers and substitution groups connect to these linkers. All of the compounds were evaluated against *L. major* with MTT assay. It seems that in these compounds c-f (Fig. 1), the type of aryl ring and the linker are the important factors for their antileishmanial activity. In the compound (c), aryl group with a piperazinyl methanone linker were the most suitable functions for antileishmanial activity.

The best compound in this series has 2-chlorophenyl substitution (IC_50_ value of 10.73μM). In the 5-nitrofuran-2-yl-1,3,4-thiadiazol-2-yl)piperazin-1-yl) benzamidine (d), the maximum antileishmanial activity observed with n-propyl substitution on benzamidine (IC_50_ value of 10μM). In other series, 5-(5-nitrofuran-2-yl)-1,3,4-thiadiazol-2-amines were synthesized by introducing N-[(1-benzyl-1*H*-1,2,3-triazol-4-yl)methyl] moiety as a new functionality on the C-2 amine of thiadiazole ring (e). The most active compound of this series has *p*-methyl substitution on phenyl ring (IC_50_ value of 12.2μM). Hence, 5-(5-nitrofuran-2-yl)-1,3,4-thiadiazole structure bearing 2-mercaptoacetamide linker (f) were prepared and displayed in vitro activity against promastigotes and amastigotes of *L. major.* In this series, 3, 4-Dimethoxyphenethyl substitution was caused to suitable activity against promastigote form of *L. major* with IC_50_ value of 19.1μM. The comparison of antileishmanial activity of compounds c-f with compounds 1a-b was showed linear substitution and thiophen ring at compound 1b are the best substitutions at C-2 and C-5 positions of thiadiazole ring. The presence of aryl group on linker is not necessary for increase antileishmanaial activity. However, in the compounds with aryl substitution, type of this substitution affected the antileishmanial activity.

Furthermore, the compounds without aryl substitution were synthesized with a simple reaction (Fig. 2) and their activity was compared with their corresponding analogues. As shown in Table 1, the substitution of the linear hydroxypropyl in the C-2 position of 1,3,4-thiadiazole ring increased the in vitro activity against promastigotes whereas the attachment of cyclic group of piperidin-4-ol exhibited lower antipromastigote activity. Linear substitution 1a-b seems to have played an important role in the mode of action of these compounds whilst cyclic substitution 5a-b has caused a steric hindrance around the C-2 position. It may have prevented the binding of the compounds to the parasitic macromolecular target. However, 5-nitrothiophene derivatives 1b and 5b with IC_50_ values of 3 and 27μM were more active than the corresponding 5-nitofuran analogues and, as such, these findings confirmed the fact that thiophen analogues were more potent than furan analogues at compounds 1a-b and 5a-b.

Besides, thiophen analogues exhibited better antiamastigote activity compared to furan analogues. Concerning low potency of compounds 5a-b compared with their linear analogues, it is suggested that likewise piperidinol ring in the 2-position of thiadiazole ring may be responsible for decreased interaction between ligand and target macromolecule in amastigote form.

The differences in the superimposition studies can help to explain why the target compounds 5a-b have lower activity. The compounds 1a and 1b have linear substitution with free rotation around single bond. However, the compounds 5a and 5b have rigid cyclic substitution (4-piperidinol) that can cause a steric hindrance around the C-2 position and, therefore, interaction between the compounds and parasitic macromolecular targets affect.

## Conclusion

Overall, the strategic positioning of substitution of linear hydroxypropyl can offer additional binding interactions probably resulting in the better binding profile of these compounds, based on the discussion above. Finally, experimental and computational investigations confirm that the cyclic derivatives 5a-b have lower activity than linear analogues 1a-b due to steric hindrance.
